# Clinical expression of Menkes disease in females with normal karyotype

**DOI:** 10.1186/1750-1172-7-6

**Published:** 2012-01-22

**Authors:** Lisbeth Birk Møller, Malgorzata Lenartowicz, Marie-Therese Zabot, Arnaud Josiane, Lydie Burglen, Chris Bennett, Daniel Riconda, Richard Fisher, Sandra Janssens, Shehla Mohammed, Margreet Ausems, Zeynep Tümer, Nina Horn, Thomas G Jensen

**Affiliations:** 1 Center of Applied Human Genetics, Kennedy Center, Gl. Landevej 7, 2600 Glostrup, Denmark; 2 Department of Genetics and Evolution, Institute of Zoology, Jagiellonian University, Ingardena 6, 30-060 Krakow, Poland; 3 Cellular Biotechnology center - GHE - Hospices Civils de Lyon - France; 4 Grenoble University Hospital, BP217, 38043 Grenoble cedex 9, France and INSERM U884, 38041 Grenoble, France; 5 Service de génétique et Centre de référence des maladies congénitales du cervelet, APHP, Hôpital Trousseau, Paris, France; 6 Yorkshire Regional Clinical Genetics Service, Ward 10, Floor 3, Chapel Allerton Hospital, Harehills Lane, Leeds LS7 4SA UK; 7 Hughes Center for Fetal Diagnostics Winnie Palmer Hospital, 83W. Miller St. Orlando, Florida, USA; 8 Teesside Genetics Unit, Northern Genetics Service, James Cook University Hospital, Marton Road, Middlesbrough TS4 3BW, UK; 9 Centre for Medical Genetics, Ghent University Hospital, Ghent, Belgium; 10 Clinical Genetics, 7th Floor Borough Wing, Guy's Hospital. London SE1 9RT, UK; 11 Department of Medical Genetics, University Medical Center Utrecht, PO Box 85090, 3508 AB Utrecht, The Netherlands; 12 Department of Human Genetics, University of Aarhus, Denmark

## Abstract

**Background:**

Menkes Disease (MD) is a rare X-linked recessive fatal neurodegenerative disorder caused by mutations in the *ATP7A *gene, and most patients are males. Female carriers are mosaics of wild-type and mutant cells due to the random X inactivation, and they are rarely affected. In the largest cohort of MD patients reported so far which consists of 517 families we identified 9 neurologically affected carriers with normal karyotypes.

**Methods:**

We investigated at-risk females for mutations in the *ATP7A *gene by sequencing or by multiplex ligation-dependent probe amplification (MLPA). We analyzed the X-inactivation pattern in affected female carriers, unaffected female carriers and non-carrier females as controls, using the human androgen-receptor gene methylation assay (*HUMAR*).

**Results:**

The clinical symptoms of affected females are generally milder than those of affected boys with the same mutations. While a skewed inactivation of the X-chromosome which harbours the mutation was observed in 94% of 49 investigated unaffected carriers, a more varied pattern was observed in the affected carriers. Of 9 investigated affected females, preferential silencing of the normal X-chromosome was observed in 4, preferential X-inactivation of the mutant X chromosome in 2, an even X-inactivation pattern in 1, and an inconclusive pattern in 2. The X-inactivation pattern correlates with the degree of mental retardation in the affected females. Eighty-one percent of 32 investigated females in the control group had moderately skewed or an even X-inactivation pattern.

**Conclusion:**

The X- inactivation pattern alone cannot be used to predict the phenotypic outcome in female carriers, as even those with skewed X-inactivation of the X-chromosome harbouring the mutation might have neurological symptoms.

## Background

Menkes Disease (MD [MIM 309400]) is a rare X-linked recessive disorder, with an incidence of about 1:298.000 [1] and is caused by mutations in the gene *ATP7A *[MIM 300011]. This gene encodes a copper transporting P-type ATPase, essential for the release of dietary copper from the intestine to the body, including the brain. The clinical features of MD are consequences of an impaired copper distribution and the malfunction of a large number of copper-requiring enzymes [2]. Classical MD is characterized by mental retardation, hypothermia, seizures, cutis laxa, hypo-pigmentation, abnormal hair (kinky hair or pili torti), and decreased serum ceruloplasmin levels [3].

The majority of Menkes patients are males and, to our knowledge, only 9 affected females have been described so far [4-10]. Five of these patients had X-autosomal translocations [5-7,9,10] one had 45X/46XX mosaicism [4], one had an unknown karyotype [8], and two patients had normal karyotypes [4]. Skewed X-inactivation has been reported previously in unaffected female carriers [11], but to our knowledge, X-inactivation has not previously been investigated in females who are affected with MD, but have a normal karyotype. This report includes the clinical description and molecular characterization of 9 affected females with normal karyotypes. Furthermore, we compare the X-inactivation pattern in the disease-manifesting carriers with the pattern in 49 asymptomatic MD carriers and the pattern in 32 normal females with no *ATP7A *mutation.

## Methods

### Subjects

In a cohort of 517 unrelated families referred to the Kennedy Center in Denmark for genetic confirmation of MD, we found 10 manifesting carriers. One patient with 45X/46XX mosaicism has been described previously [4] and will not be described further here. Nine of the affected carriers, F1-F9, have normal karyotypes. The females were born in Europe (United Kingdom, France or Germany) or United States from 1969 to 2002. The clinical phenotypes of patients F1 and F2 have been described previously [4,12].

Patient F1 (family, 9529) was born at 32 weeks gestation. Neonatally, sepsis and hyperbilirubinemia were suspected because of prematurity and a peculiar face was noted. At 31/2-4 months old: Ceruloplasmin: 0.14 g/l (normal: 0.15-0.60), S-Copper: 2 μmol/l (normal: 10-26 μmol/l). She had pneumonia, and was subsequently hospitalized several times due to other recurrent infections. She had stiff, coarse and pili torti hair, an enlarged liver and myoclonic jerks. The EEG was abnormal. At 5 years of age: Her motor development had reached the level of a 11/2-year-old child. She could only express single words, but her capacity for understanding language was better. A liver biopsy revealed a copper content in the lowest end of the normal range: 20 μg/g (normal: 20-45 μg/g), and an intestinal biopsy revealed an increased copper content: 47 μg/g (normal: 10-20 μg/g). Increased ^64^Cu uptake was observed in amniotic cells (from 16^th ^week of gestation) and in fibroblasts when she was 4 years old: 52.3 ng ^64^Cu/mg protein/20 hours, (normal: 11.5-26.7 ng). Her brother suffered from classical MD and died at 6 months of age. Two heterozygous sisters (F1-S1 and F1-S2), both also born prematurely, had no symptoms of MD.

Patient F2 (family, 93232) was born at 38 weeks gestation. Neonatally, no problems were observed. At 11 months: She was hospitalized because of severe muscular hypotonia and failure to thrive. She had myoclonic seizures, tremor and ataxia, and suffered from orthostatic hypotension. The EEG revealed numerous rapid rhythmic waves and small 4- slow bilateral overlapping waves. Pili torti of the hair was noted at the time of the diagnosis, but is no longer present. She had left-sided talipes and osteoporosis. There were recurrent episodes of infections, especially otitis during childhood. The skin was dry and joints loose during infancy and childhood. Deafness was proved at the age of 2 years. Ceruloplasmin: 0.35 g/l (normal: 0.15-0.60), S-copper: 978 μg/l (normal: 850 - 1650 μg/l) at 5 years. Increased ^64^Cu uptake and retention in fibroblasts: 87.7 ng ^64^Cu/mg protein/20 hours and 66% retention, (normal: 11.5-26.7 ng uptake and 9.7-22.7% retention). During childhood and adolescence she went to a special-needs school, but her mental retardation remains significant. She is unable to read and write and does not have any language. She is not able to walk without aid but can walk a maximum of 50 meters with the help of a walker and requires assistance to use of a wheel-chair. Due to a dysmetry of the arms, self-feeding is difficult. At 34 years of age she lives in a center for disabled people. No copper treatment was given. There is, to our knowledge, no affected male in this family.

Patient F3 (family, 9228). In the first months of life, she suffered from frequent diarrhoea and motor development delay. MRI (at about 1 year) revealed cerebral and particularly cerebellar atrophy. She was unable to hold her head up at 7 months and unable to sit up at 14 months. The hair was sparse with pili torti. Copper histidine was administered when she was 21 months. Prior to treatment her psychomotor development was retarded, and she had significant feeding difficulties with loss of weight. Following treatment, her psychomotor function improved and weight was gained rapidly. The ceruloplasmin level was below normal at 13 months, 1.5 μmol/l (normal: 2-4.30), but it rose to normal after copper treatment. She suffered from amyotrophy and dysmetria, and had frequent respiratory infections until age 10. She is mentally retarded and attends a special school. Her development at 9 years corresponded to that of a 21/2-year-old. She has never been able to walk independently, but is able to move around on her bottom. At the age of 12 years, she has no language, but communicates using pictograms. Increased ^64^Cu uptake and retention in fibroblasts: 122 ng ^64^Cu/mg protein/20 hours and 60.2% retention, (normal: 11.5-26.7 ng uptake and 9.7-22.7% retention). Her maternal uncle suffered from classical MD and died at the age of 18 months.

Patient F4 (family, 9229). She is only mildly affected. She has a slightly high arched palate, narrow thorax and slightly dry skin. The hair is thick dark, not typical of MD patients. She was able to sit up at 9 months and to walk at 3 years. She had some problems with language and mild learning difficulties, for which she was at a special school. Copper treatment has never been given. At 11 years of age: Ceruloplasmin: 0.35 g/l (normal: 0.2-0.4 g/l) and S-Copper: 19 μmol/l (normal: 12-25 μmol/l) were normal. Increased ^64^Cu uptake performed on amniotic cells from 16^th ^week of gestation, and on fibroblasts when she was 22 years old: 40.1 ng^64^Cu/mg protein/20 hours and 38.5% retention, (normal: 11.5-26.7 ng uptake and 9.7-22.7% retention). Her brother suffered from classical MD and died at the age of 8 months.

Patient F5 (family, 91284). She was able to sit up at 16 months, and to walk without support at 2 years. She could talk at 3 years, but has significant learning difficulties (IQ 64, 10 years old). Ceruloplasmin: 37.8 mg/dl (normal: 20-60 mg/dl) S-Copper: 114 μg/dl (normal: 60-180 mg/dl). The hair was brittle and sparse with pili torti. Her EEG was abnormal when she was 10 years old. Her mother has also mild learning difficulties. Her brother suffered from a slightly milder form of MD and died at the age of 7 years and 8 months.

Patient F6 (family, 92209). There were no early neonatal problems. She was subsequently noted to have short fine hair, a large fontanelle, and low muscular tone. The skin is dry and hypopigmented, and she has severe developmental delay. No copper treatment was given. Increased ^64^Cu uptake and retention in fibroblasts: 66.2 ng^64^Cu/mg protein/20 hours uptake with 45% retention, (normal: 11.5-26.7 ng uptake and 9.7-22.7% retention). Her brother suffered from classical MD and died at 21/2 years of age.

Patient F7 (family, 92283). She has severe mental and motor retardation. She had seizures until the age of 4. At the age of 41 years old she was in residential care. She is not able to walk. The hair is thin, fragile, and the skin dry. Her brother suffered from classical MD and died at 27 months. Slightly increased ^64^Cu uptake and retention in fibroblasts: 34 ng ^64^Cu/mg protein/20 hours and 30.7% retention, (normal: 11.5-26.7 ng uptake and 9.7-22.7% retention).

Patient F8 (family, 92292). She has blondish-brown hair that is moderately coarse. At the age of 14 years she was mildly mentally retarded. In addition, she had mild hypopigmentation and prominent areas of demarcated hypopigmentation all over her body but most pronounced over her abdomen, buttocks and left thigh. The vessels are tortuous. ^64^Cu uptake was normal but retention slightly increased: 22 ng ^64^Cu/mg protein/20 hours with 25-43% retention, (normal: 11.5-26.7 ng uptake and 9.7-22.7% retention). Her brother suffered from classical MD and died 25 months old. The mother and half-sister (F8-S1) are also carriers of the mutation in the *ATP7A *genes and have some signs of MD. The two sisters and the brother all have different fathers. The mother and the sister are both neurologically normal, but they both have some skin hypopigmentation, especially the sister who has also tortuous vessels, low serum copper and ceruloplasmin.

Patient F9 (family, 94228). She had mild psychomotor delay and was at the age of 29 years mildly mentally handicapped with an IQ of 83. At the age of 2 years, she suffered from convulsions. At the age of 13 she had skeletal changes (lumbosacral lordosis, genu valgum, flat feet, arachnodactyly), and ataxia. The ^64^Cu uptake in fibroblasts was slightly increased: 28.4 ng ^64^Cu/mg protein/20 hours and a clearly increased retention: 33.5% (normal: 11.5-26.7 ng uptake and 9.7-22.7% retention). No treatment was given. Her brother suffered from classical MD but received copper treatment and lived until the age of 71/2 years. At the age of 29 years she had a "professional capacities certificate" and is working. Her sister (F9-S1), also a carrier of the mutation, has mild symptoms, with an IQ slightly below normal.

### Investigation of carrier status

The carrier status of the female family members was determined by testing for the identified family-specific mutation in *ATP7A *by either sequencing or multiplex ligation-dependent probe amplification analysis (MLPA) (Salsa P104 kit, MRC, Amsterdam, Holland), depending on the type of mutation.

### DNA and RNA isolation and cDNA synthesis

Genomic DNA was prepared from peripheral blood lymphocytes, Epstein-Barr virus transformed lymphocytes, or cultured fibroblasts with the NaCl extraction method [13]. RNAeasy was used for RNA isolation (QIAgen, Bothell, WA) and single-stranded cDNA was synthesized using the High-Capacity cDNA Archive Kit (Applied Biosystems, Foster, CA).

### X-inactivation pattern

The methylation status of the human androgen receptor (*HUMARA*) gene was used as an indicator for X-chromosome inactivation [14,15]. The amplified products were analysed using an ABI 3130XL Genetic Analyzer, and the ratio between the signal intensities of the two alleles was calculated using GeneMapper 3.0 software (Applied Biosystems, Foster, CA). For each female, the ratio between signals from the two alleles in the undigested sample was used as a correction factor. DNA from an affected male from the same family was included whenever possible in order to reveal the allelic AR variant on the mutant X-chromosome.

### Quantitation of ATP7A transcript

Real-time PCR amplification of *ATP7A *transcripts from fibroblasts from the patient F2 was carried out on cDNA with an ABI7300 Genetic Analyzer in accordance with the manufacturer's instructions (Applied Biosystems, Foster, CA) as described previously [16]. We used FAM-labelled Taq-Man probes and primers that anneal to the junction between exon 22/exon 23 and exon 1/exon 2 in *ATP7A*, respectively (Applied Biosystems assay numbers: Hs00921963_m1 and Hs00921966_m1). A VIC-probe and primers to human GADPH (Applied Biosystems assay numbers: 4326317E) were used as an endogenous control.

### Copper Uptake

Copper uptake and retention in amniotic cells and fibroblasts was performed as described previously [17].

## Results

### The Menkes gene is controlled by X-inactivation

**Figure 1:**
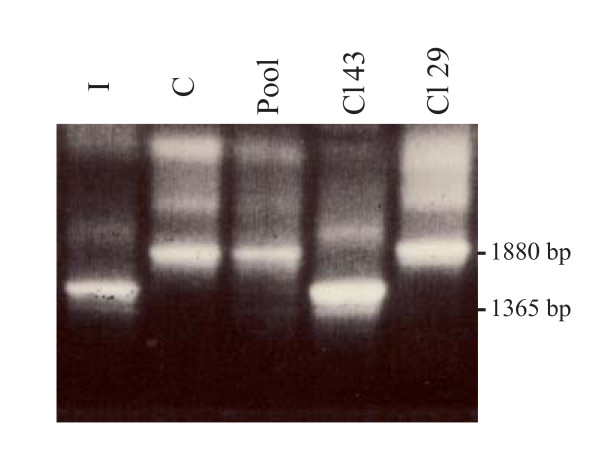
***ATP7A *transcripts in fibroblasts from a heterozygous female with the mutation c.1554-?_2172 +?del (Ex6_9del)**. Clone 43 which is characterized by increased copper accumulation and retention (63.4 ng ^64^Cu/mg protein/20 hours and 61.1% retention) expresses only the mutant transcripts (1365 bp, missing exon 6-9). In contrast clone 29, characterized by normal copper uptake and retention (11.2ng ^64^Cu/mg protein/20 hours and 11.3% retention) expresses only wild-type transcripts (1880 bp). In uncloned cell populations with normal copper uptake and retention (23.1 ng ^64^Cu/mg protein/20 hours and 11.3% retention) from the same female, only normal wild-type transcripts were detected. PCR amplification of a cDNA fragment from exon 4 to exon 10 was performed by nested PCR with the primer-pair (5'-caaaagcagcccaagtacctc-3'/5'-ggtggttgccagcacaatcagt-3') followed by the primer-pair (5'-cagaagggtcacagcaaagg-3/'5'-ggtggttgccagcacaatcagt-3). I: index (D98-34147H), affected male with the mutation Ex6_9del in *ATP7A*. C: a control sample (D98-35541H). Pool: uncloned pool from the heterozygous female (D98-35540H). Cl43: Clone 43. Cl29: Clone 29. The PCR products were separated on a 2% agarose gel and visualized with ethidium bromide.

In males, fibroblasts with a mutation in the *ATP7A *gene accumulate and retain more ^64^Cu than normal cells [17]. Heterozygous females are expected to be mosaic for *ATP7A *gene expression with one population of cells expressing the wild-type *ATP7A *gene and the other population expressing the mutated *ATP7A *gene due to random X-chromosome inactivation [18]. This has previously been shown biochemically by isolating two different cell populations (clones) from a pool of fibroblasts from a heterozygous female; one cell population accumulated a large amount of copper, just like cells from affected males, while the other cell population behaved like normal control cells [18]. We confirmed this inactivation pattern by investigating the expressed allele from two such isolated clones. Using RT-PCR analysis, we found that the cell clone with the increased copper uptake expressed mutant *ATP7A *transcripts, whereas the cell clone with the normal copper uptake expressed wild-type transcripts (Figure 1).

### Identification of mutations in affected female carriers

*ATP7A *mutations were identified in all 9 females, except for F2 (Table 1). The coding exons of *ATP7A *in F2 were screened for mutations on genomic DNA, but no mutations could be identified. In this patient the *ATP7A *transcript was below the detection limit, in contrast to the normal amount of the endogenous control *GADPH *transcript (not shown).

**Table 1:** Summary of clinical, biochemical and genetic findings in manifesting carriers

	Patients								
Symptoms	F1 (9529)^d^	F2 (93232)	F3 (9228)	F4 (9229)	F5 (91284)	F6 (92209)	F7 (92283)	F8(92292)	F9 (94228)
**Mental retardation**	++ 5 years old, could only express single words	+++ 34 years old, unable to read, write, talk	+++ 9 years old, no language	+ 22 years old, mild mental retardation	++ 10 years old, significant learning difficulties	+++ 2 years old, severe mental retardation	+++ 41 years old, severe mental retardation	+ 14 years old, mild mental retardation	+ 29 years old, mild mental retardation

**Motor retardation**	++	+++	+++	+	No	+	+		No

**Convulsions**	+	+	No	No	No		+		No

**Ataxia**	+	+	+		No				+

**Hypotonia**	+	+	+			+			No

**Hypothermia**	+	No	+	No	No		No		

**Failure to thrive**	+	+	+	No	No				

**Diarrhoea**	-	No	+	No	No		No		

**Dry skin**	+	+	+	+	+	+	+		+

**Skeletal Changes**	+	+	+						

**Occipital horns**			+				No		

**Loose joints**		+	+	No	No		No		+

**Cutis laxa**		No	+	No	No				+

**Abnormal hair**	+	+	+	No	+	+	+	+	

**Hypopigmentation**	+	No	+		+	+	No	+	

**Born, gestation week**	32^nd^	38^nd^	35^nd^	35^nd^	40^nd^	38^nd^	36^nd^		

**Recurrent infections**	+	+	+				No		

**Reduced serum Copper/ceruloplasmin**	+/+	No/No	+/+	No/No	No/No			+/+	No/No

**Increased Cu uptake/retention**	+/NA	+/+	+/+	+/+	NA/NA	+/+	+/+	No/+	+/+

**Mutation**	c.1946 + 5G > A (IVS8 + 5G > A)	Unknown^b^	c.1554-?_ 1707 +?del (Ex6del)	c.1554-?_ 2172 +?del (Ex6_9del)	c.4123 + 5G > A (IVS21 + 5G > A)	c.2179G > A (p.G727R)	c.2383C > A (p.R795X)	c.-22-?del (Ex1del)	c.532G > T (p.E178X)

**X-inactivation ratio(M:N)^a ^**	24:76	0:100^c^	I.C.	80:20	51:49	4:96	0:100	I.C	100:0

Empty squares indicate that the information is not available. ^a^Ratio denote the percentage of cells with the X-chromosome bearing the ATP7A mutation inactivated (M): percentage of cells with the normal chromosome inactivated (N). NA; not analysed. ^b^The mutation is not identified. Quantization of ATP7A mRNA transcript revealed that the level was below the detection limit. ^c^We assume that the normal allele is inactivated based on the absent of ATP7A transcript. I.C. Inconclusive. ^d^F1 has two heterozygous sisters both also born prematurely, but with no symptoms of Menkes disease.

Based on the clinical phenotypes and the carrier status we suggest that all 9 clinically affected females are suffering from MD (F1-F9). This is supported by the fact that the copper uptake and/or retention were increased in all affected females (not measured in patient F5). Serum copper and ceruloplasmin were not reduced in all 9 patients, but the levels can be normal - also in affected males with mild symptoms (Table 1).

### X-inactivation patterns in affected female carriers

A possible association between skewed X-inactivation and disease severity was investigated by measuring the X-inactivation patterns in the 9 affected females (Table 2). One female (F9) had a highly skewed inactivation of the mutant X-chromosome (ratio = 100:0), and three females (F6, F7, and probably F2) had highly skewed inactivation of the normal X-chromosome (ratio ≤ 4:96). Two females had moderately skewed inactivation of either the mutant X-chromosome (F4) (ratio = 80:20) or the normal X-chromosome (F1) (ratio = 24:76), and one female had an even X-inactivation pattern (F5). The X-inactivation assay was inconclusive for two females (F3, F8) due to homozygosity of the AR polymorphism.

**Table 2:** X-inactivation pattern in affected females and family members

Family, mutation type	Affected females (n = 9) Inactivation mutant X:normal X	Unaffected carriers (n = 15) Inactivation mutant X:normal X	Non-carriers (n = 4) Activity of X1:X2
**F1, splicing intron 8**	24:76% (3426-85F)	2 people:	3 people: 95:5% (M85-3427F);
		100:0% (79185F);	70:30%.(3494-85F);
		98:2% (78985F)^a^	70:30% (D92-5215T)

**F2, unknown**	0:100% (D92-6267F)		
	(based on Real time RT-PCR results)^b^		

**F3, exon 6del**	Inconclusive (D00-43654F)	1 person: 90:10% (5974B)	

**F4, exon 6_9del**	80:20%; 78:22% (D91-3753T; D67859F)	6 people: 98:2% (AM280F);100:0%(1161T);	1 person: 100:0% (67807F)
		100:0% (67803F); 100:0% (67802F);	
		73:27% (44505T); 96:4% (41569B)	

**F5, splicing intron 21**	51:49% (33744B)	1 person:78:22% (39012B)^c^	

**F6, missense exon 10**	4:96% (59135F)		

**F7, nonsense exon 10**	0:100% (D92-5810T)	2 people:	
		92:8%;100:0% (D91-1195T; D95-24179F);	
		100:0% (D92-5613T)	

**F8, exon 1del**	Inconclusive (D92-7418F)	1 person:100:0% (D94-1503F)^d^	

**F9, nonsense exon 3**	100:0%(23508F)	2 people:	
		100:0% (23509F)^e^;	
		74:26%(41882F)	

DNA extracted from B: blood sample; T: transformed lymphocytes; F: cultured fibroblasts.

^a^Sister F1-S1. ^b^The mother of F2 showed also highly skewed X-inactivation pattern, but with the opposite X chromosome active.

^c^Mother of F5. ^d^Sister F8-S1.^e^Sister F9-S1.

The clinical phenotypes, biochemical and molecular findings of the 9 clinically affected carriers (F1-F9) are summarized in Table 1.

### X-inactivation patterns of unaffected female carriers

For comparison, the X-inactivation patterns of 49 unaffected female carriers were measured. The first sub-group was composed of 15 women from the same families as the manifesting carriers. (Table 2). The second sub-group consisted of 34 unaffected carriers from other Menkes families (Table 3). A very high fraction of the unaffected carriers (69%, 34 females out of 49) had highly skewed inactivation of the mutant X-chromosome (ratio ≥ 90:10), whereas a minor fraction (24%, 12 females out of 49) had a more moderate inactivation (90:10 ≥ ratio ≥ 70:30) of the mutant X-chromosome, and only three females exhibited random X-inactivation. These results seem to be independent of both the DNA-source (lymphocytes, transformed lymphocytes or fibroblasts), family and of the type of mutation in the *ATP7A *gene (Table 2 and 3).

**Table 3:** X-inactivation status of members of other families affected with MD

Family, mutation type	**Unaffected carriers (n = 34)**. Inactivation mutant X:normal X	Non-carriers (n = 28) Activity of X1:X2
**942, 1 bp dup exon 10**	2 people: 93:7% (AM45F); 91:9% (AM44F)	1 person: 46:54% (AM93F)

**91207, nonsense exon 18**	3 people: 99-100:1-0% (28386B; 28384B; 28356B).	1 person: 83:17% (28388B)
	1 person: 54:46% (36054B)	1 person: 73:27% (36052B)
		2 people: 58:42% (28389B); 58:42% (36053B)

**93210, 2 bp dup exon 21**	2 people: 90:10% (12499B); 98:2% (12496B)	

**9129, splicing intron 9**	1 person: 100:0% (92-5408T)	2. people: 68:32% (91-581T); 65:35% (91-420T)
	1 person: 86:14% (91-578T)	

**93220, splicing intron 6**	2 people: 100:0%(12809F); 100:0% (12810F)	1 person: 90:10% (12746F)
		1 person: 60:40% (12745F)

**94252, missense exon 10**	2 people: 94:6% (29620B); 100:0% (29623B)	1 person: 80:20% (29619B)
	1 person: 80:20% (29617B)	2 people: 51:49% (29621B); 56:44% (29624B)

**95254, missense exon 10**	2 people: 98:2% (48625B); 91:8% (48627B)	1 person: 61:39% (48626B)
	1 person: 74:26% (46672B)	

**94267, missense exon 12**	2 people: 75:25% (37443B); 84:16% (37435B)	1 person: 74:26% (37437B)
		2 people: 51:49%(3744B2); 52:48% (37440B)

**9328, missense exon 12**	1 person: 100:0% (2429T)	1 person: 78:22% (2351T)
		1 person: 52:48% (2350T)

**91204, exon 3_4del**	2 people: 99:1% (11559F; 11761F)	1 person: 91:9% (11864B)
		1 person: 73:27% (11863B)

**96249, exon 8_9del**	2 people: 87:13% (65547B); 83:17% (63056B)	1 person: 73:27% (67297B)
	1 person: 78:22% (65646B)	

**9727, exon 12_15 del**	1 person: 81:19% (78773B)	1 person: 86:14% (29898F)
		1 person: 73:27% (32531B)
		1 person: 62:38% (32532B)

**91262, exon 13_17del**	3 people: 99-100:1-0% (AM261F, AM264F, 51646F)	

**9720, missense exon 20**	1 person: 92:8% (67806F)	1 person: 53:47% (67884F)
	2 people: 48:52% (67808F); 37:63% (67804F)	

**91269, exon 13_17del**	1.person:100:0% (D91-538T)	2 people:100:0% (553T); 100:0% (10340T)
		1 person: 60:40% (38179F)

DNA extracted from B: blood sample; T: transformed lymphocytes; F: cultured fibroblasts.

### X-inactivation patterns of normal non-carrier females

The X-inactivation patterns of 32 non-carrier females from the same Menkes families revealed that 6 individuals had highly skewed (ratio ≥ 90:10) and 11 individuals had moderately skewed (90:10 ≥ ratio ≥ 70:30) X-inactivation patterns. About half of the non-carriers (47%, 15 of 32) had an almost even X-inactivation pattern (70:30 ≥ ratio ≥ 50:50) (Table 2 and 3).

## Discussion

Affected carriers of MD are rare. In a total of 517 families we know of 9 clearly affected females. However, we know also of mildly affected females who have not been characterized as manifesting carriers. These mildly affected females had pili torti, hypopigmentation of the skin or other mild symptoms of MD. The total number of females with any symptoms of MD is unknown.

Due to random X-inactivation, females are mosaic for X-linked gene expression. On average, approximately 50% of the cells express genes from the paternal X-chromosome and the other 50% express genes from the maternal X-chromosome [19]. Three different mechanisms for skewed X-inactivation patterns have been suggested [20]: 1) Skewed X-inactivation by chance. 2) A genetic defect in the X-inactivation process. 3) Post-inactivation growth advantage/disadvantage for cells with a mutation on the X-chromosome. It has been shown that several X-linked mental retardation disorders have a strong association with skewed X-chromosome inactivation in carrier females. The mechanism is however unknown [21].

The observation that most unaffected carriers have an inactivation of the mutant X-chromosome concurs with the recent paper by Desai et al. [11], in which complete inactivation of the mutant X-chromosome was observed in one Menkes family with 6 heterozygous females. We also observed skewed inactivation of the mutant X chromosme in most unaffected carriers. We found highly skewed inactivation (≥ 90:10) of the mutant X-chromosome in 69% of the asymptomatic carriers, and moderately skewed inactivation of the mutant X-chromosome in 24% of the carriers. Only 6% exhibited an even inactivation and none of the female exhibited skewed inactivation of the normal X-chromosome. Investigation of the manifesting carriers revealed a large variation in the observed X-inactivation pattern. Although more than half of the affected females (57%, 4 out of 7) had skewed inactivation of the normal X-chromosome, complete inactivation of the mutated X-chromosome was also observed in one female, whereas an even X-inactivation pattern was found in another. Finally, about 19% of the normal females without any mutation in the *ATP7A *gene exhibited highly skewed X-inactivation. In conclusion, skewed X-inactivation of the mutant X chromosome was noted in the majority of asymptomatic carriers whereas skewed X-inactivation of the normal X-chromosome was noted in the majority of the affected females. Thus although a clear tendency was observed, a number of exceptions from this tendency were observed.

X-inactivation is influenced by several factors and the X-inactivation in DNA from blood samples, cultured EBV transformed lymphocytes or fibroblast cultures may not reflect the pattern in the rest of the body. In addition, the risk of a recombination between the *ATP7A *locus and the *AR *locus during meiosis could lead to a faulty interpretation. The distance between the two loci corresponds to a recombination frequency of about 12%. Furthermore, the pattern obtained from a cell culture might depend on the number of passages. Long-term culture can affect the X-inactivation pattern (own unpublished observation).

The symptoms of MD were milder in all 9 analyzed manifesting carriers than in affected males with the same mutations. Whereas affected males from the same families had the classical form of MD (except the brother of F5 who had a slightly milder form of MD with prolonged survival), the affected females survive longer, and in general they exhibit milder symptoms. Five female cases of MD carrying X-autosome translocations have been described previously. Although the clinical symptoms of these females are generally more severe than the symptoms of the affected females with normal karyotypes described here, the clinical features were, at least also for the majority of these female patients, milder than those of the affected boys [9,10]. Studies of late replicating X performed in three of the five patients with X-autosome translocations revealed that the normal X-chromosome was inactive, at least in the majority of the analyzed cells [7,9,10].

The milder phenotypes observed in females with normal karyotypes could be attributed to higher expression levels of the normal *ATP7A *gene. The degree of X-inactivation of the normal X-chromosome correlated with the degree of mental retardation. Highly skewed inactivation of the normal X-chromosome was observed in affected females with severe mental retardation (F6, F7, and probably F2), whereas highly skewed inactivation of the mutant X-chromosome was only observed in one female with mild mental retardation (F9).

It has been shown that certain X-linked genes, among them the *ATP7A *gene, escape X-inactivation to a certain degree [22]. This phenomenon might affect the phenotype in both group of affected females, those with a balanced X-autosomal translocation and those with a normal karyotype, in which the normal X-chromosome is inactivated. The milder phenotypes, compared to those of affected males might be attributed to expression of normal *ATP7A *transcript from the inactivated normal X-chromosome.

## Conclusions

Few females are affected with MD, and their clinical symptoms are generally milder than those of affected boys with the same mutations. Although the X-inactivation pattern correlates somewhat with presence or absence of MD signs in female carriers, it does not sufficiently explain the observed phenotypes in all of them, as even those with a skewed inactivation of the X-chromosome that harboured the mutation, might have neurological symptoms. Thus, the X-inactivation pattern alone can not be used to predict the phenotypic outcome in female carriers.

## List of abbreviations

MD: Menkes disease; MLPA: Multiplex ligation-dependent probe amplification analysis; *HUMARA*: human androgen receptor.

## Competing interests

The authors declare that they have no competing interests.

## Authors' contributions

Generation and analysis of the clinical data: MTZ, AJ, LB, CB, DR, RF, SJ, SM, MA. Mutation identification: LBM, ZT. X-inactivation analysis: LBM. Study concept: LBM. Manuscript draft: LBM. Substantial participation in the design of the project and writing the paper: TGJ, ML, NH, MTZ, AJ, LB, SM. Copper uptake and retention: NH, LBM. All authors participated in the writing of this paper in the context of their individual expertise, and all have read and approved the final version of the article.
